# RETRACTION: Effect of Wound Protectors in Reducing the Incidence of Surgical Site Wound Infection in Lower Gastrointestinal Surgery: A Meta‐Analysis

**DOI:** 10.1111/iwj.70465

**Published:** 2025-04-02

## Abstract

**RETRACTION:** MaoL. , ZhouS. , LiaoJ. , ZhouX. , and WangJ. , “Effect of Wound Protectors in Reducing the Incidence of Surgical Site Wound Infection in Lower Gastrointestinal Surgery: A Meta‐Analysis,” International Wound Journal 20, no. 3 (2023): 813–821, 10.1111/iwj.13928.36117245 PMC9927917

The above article, published online on 18 September 2022, in Wiley Online Library (http://onlinelibrary.wiley.com/), has been retracted by agreement between the journal Editor in Chief, Professor Keith Harding; and John Wiley & Sons Ltd. Following an investigation by the publisher, all parties have concluded that this article was accepted solely on the basis of a compromised peer review process. The editors have therefore decided to retract the article. The authors agree with the retraction. The authors agree with the retraction.

**RETRACTION:** L. Mao , S. Zhou , J. Liao , X. Zhou , and J. Wang , “Effect of Wound Protectors in Reducing the Incidence of Surgical Site Wound Infection in Lower Gastrointestinal Surgery: A Meta‐Analysis,” International Wound Journal 20, no. 3 (2023): 813–821, 10.1111/iwj.13928.36117245 PMC9927917

The above article, published online on 18 September 2022, in Wiley Online Library (http://onlinelibrary.wiley.com/), has been retracted by agreement between the journal Editor in Chief, Professor Keith Harding; and John Wiley & Sons Ltd. Following an investigation by the publisher, all parties have concluded that this article was accepted solely on the basis of a compromised peer review process. The editors have therefore decided to retract the article. The authors agree with the retraction. The authors agree with the retraction.

